# Antioxidants Special Issue: Peroxiredoxin 6 as a Unique Member of the Peroxiredoxin Family

**DOI:** 10.3390/antiox8040107

**Published:** 2019-04-19

**Authors:** Aron B. Fisher

**Affiliations:** Department of Physiology and the Institute for Environmental Medicine, University of Pennsylvania, Philadelphia, PA 19104, USA; abf@upenn.edu

The peroxiredoxins, first discovered about 30 years ago, are the most recently described family of ubiquitously expressed antioxidant enzymes [1,2]. These proteins have been classified into six groups (PRX1, PRX5, PRX6, PRXQ, TPx, and ahpE) that include both vertebrate and non-vertebrate forms [3]. A mammalian-only classification also recognizes six groups by expanding the PRX1 group into four closely related sub-groups (PRX1-4) plus PRX5 and PRX6. PRX6 is frequently abbreviated Prdx6, as is used in this Special Issue. Prdx6, first isolated about 25 years ago, was the last of the mammalian family of peroxiredoxins to be described and its molecular sequence was published shortly afterwards [4,5,6]. In the older literature, this enzyme also has been called 1-cys peroxiredoxin, nonselenium glutathione peroxidase (GPx), acidic Ca^2+^-independent phospholipase A_2_ (aiPLA_2_), antioxidant protein 2 (AOP2), Clara cell protein 26 (CC26), and protein p29 [7]. While Prdx6 shows sequence homology with the other PRX forms and like them functions to reduce H_2_O_2_, short chain hydroperoxides, and peroxinitrite [1,8], it also shows some important distinguishing characteristics.

The special characteristics that differentiate Prdx6 from the other PRXs include:

(1) Catalytic mechanism: All peroxiredoxins express a conserved cysteine (Cys) residue, called the peroxidatic Cys, that is oxidized by interaction with H_2_O_2_ or other oxidant substrate. The PRX 1–5 family members express a second (resolving) Cys that, in conjunction with thioredoxin, reduces the peroxidatic Cys and restores the physiologically active form. Prdx6, however, expresses only a single conserved Cys and uses glutathione (GSH) plus GSH S-transferase (GST) for reduction and resolution of its oxidized peroxidatic Cys [9];
(2) Substrate binding: Unlike other PRXs, Prdx6 can bind to phospholipids [10]. This is important for several enzymatic activities of Prdx6 (described next) that are not present in other members of the PRX family of enzymes.
(3) Phospholipid hydroperoxide reductase activity: Prdx6 is able to bind and to reduce phospholipid hydroperoxides that may be produced as a result of oxidative stress [11]. This phospholipid hydroperoxide reductase activity is analogous to the enzymatic activity of GSH peroxidase, type 4 (GPx4); the protein with the dominant reductase activity in any given tissue appears to vary with cell type [12].
(4) Phospholipid hydrolysis: Phospholipids bound to Prdx6 can be hydrolyzed at the *sn*-2 position indicating a phospholipase A_2_ (PLA_2_) activity [13];
(5) Lysophosphosphatidylcholine acyltransferase (LPCAT) activity: Prdx6 is able to acylate lysophospholipids (lysophosphatidylcholine is the primary substrate) by a transferase reaction to generate a phospholipid (phosphatidylcholine) [14].The coupling of the PLA_2_ and LPCAT activities of Prdx6 represents a major mechanism for phospholipid remodeling through hydrolysis followed by re-acylation at the *sn*-2 position [7,12].
(6) Subcellular localization: Like several other PRXs, Prdx6 is localized primarily to cytosol, but it is also the only member of the PRX family to be present in both lysosomes and lysosomal related organelles such as the lung lamellar bodies that are a site for synthesis and storage of the lung surfactant [15].

These six special characteristics of Prdx6 allow this protein to play specific and important roles in normal physiology and pathobiology including the scavenging of oxidants, the repair of peroxidized cell membranes, the turnover of lung surfactant phospholipids, and cellular signaling as mediated by reactive oxygen and nitrogen species (ROS/RNS) [12,16,17,18]. These functions of Prdx6 are postulated as important in various disease states including inflammation, acute lung injury, cancer, chronic diseases of the CNS, type II diabetes, and male infertility among others. Many of these topics are explored in depth in this special issue that includes five review articles and five articles reporting original research.

The first article in this special issue is a review by Feinstein that reports on currently available mouse models to evaluate the physiological and pathophysiological roles of Prdx6 [19]. Of special interest are the models to identify the specific roles of the GSH peroxidase vs. the PLA_2_ activities of Prdx6 using mice with C47S-Prdx6 and D140A-Prdx6 mutations. The second article by Bannitz-Fernandes et al. describes original research that, for the first time, shows the presence of PLA_2_ activity in several non-mammalian Prdx6 enzymes [20]. The original research by Shahnaj et al. in the third article of this FORUM used recombinant mammalian Prdx6 to demonstrate that hyperoxidation of the protein results in the formation of multimers [21], similar to that shown for other members of the peroxiredoxin family [22]. The fourth article, original research by Zhou et al., shows that the presence of GSH can lead to hyperoxidation of the protein in vitro, resulting in the loss of peroxidase activity but a significant increase in PLA_2_ activity at cytosolic pH; this effect was unrelated to the formation of multimers [23]. The fifth article by Allervajo and Vazquez-Medina reviews the role of Prdx6 in cell signaling with special emphasis on superoxide anion (O_2_•−) generation by NADPH oxidase (NOX2) and its important role in cellular communication [24]. Prdx6 generates lysophosphatidylcholine through its PLA_2_ activity, that results in the downstream activation of Rac, a required co-factor for the activation of NOX2. The following original research article by Fisher et al. identifies several peptides derived from the naturally occurring protein surfactant protein A (SP-A) that can inhibit the PLA_2_ activity of Prdx6 and prevent the activation of NOX2 [25]. The seventh article by Patel and Chatterjee reviews cellular signaling with focus on the endothelium [26]. The authors present evidence that the regulation of Prdx6 expression and activity is crucial to endothelial cellular homeostasis and discuss the role of Prdx6 in mediating various pathologies. One of those pathologies, Fuchs endothelial corneal dystrophy (FECD), is a leading indication for corneal endothelial transplantation as described in the subsequent article by Lovatt et al.; this report of original research is focused on the role of Prdx6 in the preservation of corneal endothelial cellular integrity [27]. The ninth article by Sharapov et al. reviews the ability of Prdx6 to protect against X-irradiation-induced injury such as that used for treatment of cancer [28]. Both exogenous Prdx6 as well as increased expression of endogenous Prdx6 provide radioprotection. The tenth and final contribution to the special issue is a review by O’Flaherty that focuses on male fertility [29]. This review postulates that Prdx6 is the primary antioxidant enzyme that protects spermatozoa from oxidative stress-associated damage. Thus, the 5 articles of new research along with the 5 review articles cover a broad spectrum of Prdx6 function in physiology and pathophysiology and will serve as a base for continued studies of this important protein.

Despite the considerable increase during the past 25 years in our knowledge of Prdx6, there remain large gaps in our understanding of its structure-function relationships and (patho)physiological roles. Although a structural mechanism to account for its ability to bind phospholipids was proposed some time ago [30], there has not been definitive confirmation of this scheme (nor an acceptable alternative proposed) despite two publications using X-ray crystallographic analysis [31,32] and another using a zero length crosslinking technique [33]. Likewise, there has not been identification of the mechanism for the marked increase in PLA_2_ activity following phosphorylation of the protein, although the Thr177 amino acid in Prdx6 has been identified as the phosphorylation site [34] and a change in protein confirmation has been shown to be required for the increased activity [35]. Another intriguing question relates to the roles of the enzymatic activities of Prdx6 in cellular function. None of the activities of Prdx6 is unique and a variety of other dedicated enzymes also can reduce H_2_O_2_, hydroperoxides, and peroxynitrite, hydrolyze phospholipids (PLA_2_ activity), and transfer acyl groups. In many cases, the impact of Prdx6 may relate to its specific tissue expression as seems to be the explanation for the predominant role of Prdx6 to reduce phospholipid hydroperoxides in the lung [12]. But, the determinants for expression of a particular enzyme in particular cells (as opposed to expression of another enzyme with similar activity) is largely unknown. With respect to the role of Prdx6 in pathophysiology, altered expression of the protein has been shown with many types of human cancers and expression levels have been shown to alter cancer growth rates as well as metastatic potential (reviewed in [13]). Altered Prdx6 expression also has been demonstrated in many types of neurodegenerative disease (reviewed in [13]). However, no reasonable mechanism has been proposed or studied related to these pathophysiologic effects of altered expression. So, the basic unresolved issues discussed above, as well as other issues that undoubtedly will be identified by future studies, indicate the need for considerable additional work to explore the structure–function relationships and the (patho)physiologic roles of this intriguing enzyme.
